# Anticipating Biopreservation Technologies that Pause Biological Time: Building Governance & Coordination Across Applications

**DOI:** 10.1017/jme.2024.129

**Published:** 2024-12-16

**Authors:** Susan M. Wolf, Timothy L. Pruett, Claire Colby McVan, Evelyn Brister, Shawneequa L. Callier, Alexander M. Capron, James F. Childress, Michele Bratcher Goodwin, Insoo Hyun, Rosario Isasi, Andrew D. Maynard, Kenneth A. Oye, Paul B. Thompson, Terrence R. Tiersch

**Affiliations:** 1 UNIVERSITY OF MINNESOTA, MINNEAPOLIS, MINNESOTA, USA.; 2 ROCHESTER INSTITUTE OF TECHNOLOGY, ROCHESTER, NEW YORK, USA.; 3 GEORGE WASHINGTON UNIVERSITY, WASHINGTON, DC, USA.; 4 UNIVERSITY OF SOUTHERN CALIFORNIA, LOS ANGELES, CALIFORNIA, USA.; 5 UNIVERSITY OF VIRGINIA, CHARLOTTESVILLE, VIRGINIA, USA.; 6 GEORGETOWN UNIVERSITY, WASHINGTON, D.C., USA.; 7 MUSEUM OF SCIENCE, BOSTON, MASSACHUSETTS, USA.; 8 UNIVERSITY OF MIAMI, MIAMI, FLORIDA, USA.; 9 ARIZONA STATE UNIVERSITY, TEMPE, ARIZONA, USA.; 10 MASSACHUSETTS INSTITUTE OF TECHNOLOGY, CAMBRIDGE, MASSACHUSETTS, USA.; 11 MICHIGAN STATE UNIVERSITY, EAST LANSING, MICHIGAN, USA.; 12 LOUISIANA STATE UNIVERSITY, BATON ROUGE, LOUISIANA, USA.

**Keywords:** Biopreservation, Cryopreservation, Governance, Ethics, Emerging Technology

## Abstract

Advanced biopreservation technologies using subzero approaches such as supercooling, partial freezing, and vitrification with reanimating techniques including nanoparticle infusion and laser rewarming are rapidly emerging as technologies with potential to radically disrupt biomedicine, research, aquaculture, and conservation. These technologies could pause biological time and facilitate large-scale banking of biomedical products including organs, tissues, and cell therapies.

## Introduction

**Figure 1:**
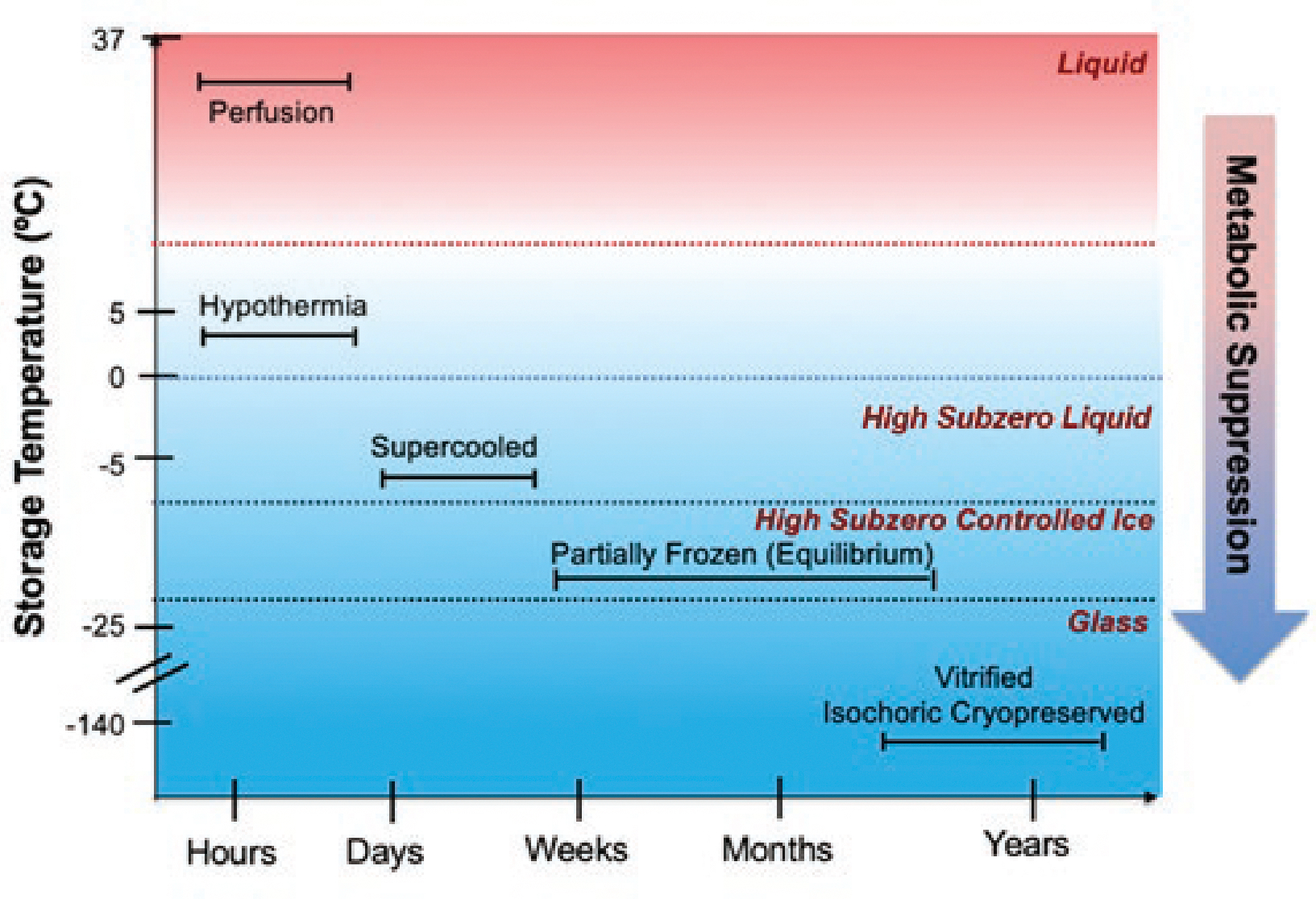
Comparing advanced biopreservation techniques for solid organs (all below 0° C) with conventional cryopreservation and perfusion (above 0° C). Note that each advanced biopreservation technique can be applied within a range of temperatures, so temperatures in this figure are approximate. Isochoric techniques are not shown because those techniques rely on manipulation of the chamber’s volume, a different dimension not shown here. (Figure used with permission from ATP-Bio)

Emerging technologies for prolonged biopreservation have the potential to radically alter practices in the biomedical and life sciences ranging from environmental conservation to health care. (See Table 1 for a list of four major categories of use for advanced biopreservation techniques.) Techniques including supercooling,^1^ partial freezing,^2^ isochoric techniques,^3^ and vitrification,^4^ combined with nanoparticle infusion and laser rewarming could halt metabolism, allow mass storage of biological materials for extended periods of time, and enable transport over large distances, including potential space travel. (See Figure 1 focusing on application to solid organs for transplantation, in order to compare advanced biopreservation with conventional cryopreservation.) Conventional cryopreservation typically uses low temperatures to slow metabolism and preserve biological materials.^5^ However, advanced biopreservation uses subzero techniques such as supercooling, partial freezing, and vitrification (sometimes in combination with isochoric techniques confining the volume of the container) in innovative ways to preserve a range of biological materials at different scales, including whole organs and organisms. These advanced biopreservation techniques can transform precious biological materials whose viability is now limited by time and geography into off-the-shelf products available on demand from large-scale repositories. The potential for benefit and harm demands attention, allow for stockpiling tissue in case of mass emergencies, and extend the life of cell therapies such as CAR-T cells used in cancer treatments.^7^ The technologies could also preserve whole organisms ranging in size from single-cell pathogens^8^ to model organisms,^9^ greatly facilitating research. Beyond health care and biomedical research, advanced preservation tech-in order to shape the coming technological transition and develop effective governance approaches.

Advanced biopreservation techniques will change human biomedicine. Technologies now in development have the potential to transform the use of biological materials, from cell therapies to tissue grafts to organ transplants.^6^ Advanced biopreservation could improve availability of transplantable donor organs, nologies could support development of aquaculture and agriculture by preserving and enabling distribution of valuable genetic lines.^10^ Finally, advanced biopreservation could act as an “insurance policy”^11^ to preserve biodiversity by banking genetic material from the wide diversity of animal species, and even permit creation of a lunar biorepository in case of catastrophe on earth.^12^

Since Giwa and colleagues reviewed the emerging field of biopreservation in biomedicine in 2017,^13^ progress has been swift. In 2019, researchers reported successfully preserving a human liver via supercooling and ex vivo machine perfusion for 27 hours.^14^ Organ vitrification is also developing rapidly, though still being perfected in pre-clinical studies. Recent work has demonstrated viable vitrification and rewarming of human pancreatic islet cells.^15^ Researchers have successfully preserved rabbit kidneys for 24–48 hours^16^ and have vitrified rat kidneys for up to 100 days with nano-rewarming and successful return to function.^17^ Clinical trials of vitrified ovarian tissue show promise in preserving fertility.^18^

Advanced preservation techniques also show great promise for non-human applications, including whole organisms, food and aquaculture, and conservation. Cryopreservation of *Cryptosporidium* offers an alternative to constantly propagating the parasite in laboratory animals.^19^ Cryopreservation and subsequent laser nanowarming of zebrafish embryos could facilitate storage of mutant and transgenic lines of the crucial model organism.^20^ Cryopreservation of germplasm from agriculturally significant species can support agricultural producers and ensure long-term species survival.^21^ In aquaculture, cryopreservation of sperm has long been possible, but new technologies such as vitrification and laser rewarming may facilitate storage of oocytes and embryos.^22^ Enhancing preservation abilities could allow easier and less resource-intense broodstock management for aquaculture producers.^23^ Advanced preservation technologies can store genetic material from animal species to preserve biodiversity.^24^ Vitrification and laser rewarming have been successfully applied to the embryos of cows, rats, rabbits, goats, sheep, domestic cats, and horses.^25^ Scientists have successfully applied isochoric vitrification techniques to coral fragments to support *ex situ* conservation of threatened coral reefs.^26^

As these technologies advance, however, oversight is lagging. Even in the heavily regulated domain of biomedical products for human application, US authorities and stakeholders are not prepared to oversee the new wave of biopreservation technologies. Organ transplantation is governed by multiple regulatory authorities in the US, with no clarity on governance of the preservation, storage, transportation, and reanimation involved in advanced biopreservation. Oversight of tissue banking is a complex interplay of federal and state rules, as well as professional society standards, with little attention as yet to the challenges of prolonged preservation on a large scale for uses ranging from patient care to mass casualty response. Increasingly sophisticated cell-based therapies are challenging the US Food and Drug Administration (FDA) to develop adequate oversight, as regulators across the world are already grappling with a $2.4 billion market for unproven cell therapies that may be ineffective or even dangerous.^27^ Advanced biopreservation technologies will not only present new processes and products, they will also demand new supply chains and facilities, including biopreservation centers, biorepositories for prolonged storage, and rewarming facilities. Successful development and application of these technologies to advance human health will require extensive stakeholder engagement and consultation, including among patients and the broader public.^28^

Non-human applications pose oversight challenges as well. Cryopreserving pathogens raises dual-use concerns, enabling research but also inviting accidental or deliberate misuse.^29^ Preserving model organisms requires the development of standard operating procedures (SOPs) to ensure uniformity and reproducibility.^30^ Applying preservation techniques to aquaculture requires regulatory improvements, public trust in biopreserved food species, and accessible technology. Realizing the conservation benefits of advanced preservation technologies will require stakeholder engagement — including in affected communities — to ensure that these technologies yield ecosystem benefit, not harm. In many cases, such as for aquatic species, the needs are diverse but generalized solutions can be developed if the problems are addressed at a community level linking different research groups and technology stakeholders. In contrast, work by multiple separate groups pursuing diverse goals, often in competition with one another, can exacerbate the governance challenge and slow down solutions.

There are also problems with addressing oversight along the technology development pipeline. Concerns at the bench-scale research level may be different from those in the subsequent processes of development, small-scale application, enterprise-level commercialization, and full industrial adoption. As such, “appropriate” governance at any single level may not transfer directly or even support transition to the next. Thoughtful consideration of the entire pipeline early in this progression could smooth transitions and support progress.

This “pacing problem” — with science outpacing development of law and broader oversight mechanisms such as consensus standards — is familiar from multiple domains of emerging technology.^31^ Governance confusion and gaps can slow innovation that could confer benefit, while leaving risks unaddressed. A range of governance approaches have been suggested across the landscape of emerging technologies.^32^ These approaches range from “hard” governance using command-and-control regulation by governmental entities, to “soft” governance by non-governmental entities such as professional societies promulgating guidelines, voluntary standards adopted by commercial companies, and normative frameworks adopted by communities.

Oversight of advanced biopreservation technologies is complicated by the breadth of techniques and applications across domains. Creating governance structures for emerging technologies that cut across sectors is a challenging task. This problem is not unique to biopreservation. Other multi-use platform technologies such as synthetic biology,^33^ artificial intelligence,^34^ and nanotechnology^35^ have raised similar issues. Different applications of multi-use technologies “may warrant different oversight regimes.”^36^ This may mean that “no single regulatory agency has the full picture of a technology or complete jurisdiction over it,”^37^ even though applications in one sector may have implications for the development of applications in another sector.^38^

Responsible research and development require anticipating the ethical, legal, and societal challenges to develop needed governance structures.^39^ Part I of this article canvasses major approaches to developing governance of emerging technologies. Part II then considers the specific oversight challenges posed by key applications of biopreservation. Part III suggests strategies to coordinate governance of biopreservation technologies across applications in order to realize benefit and control risk as the technologies evolve. Coordination should involve development of standardized terminology, protocols, and measures plus exchange across the otherwise-siloed communities in each domain of application. Coordinated governance efforts and consultation with stakeholders can begin to address the oversight challenges.

### I. Developing Oversight of Emerging Technologies

The breadth of technologies and applications under the umbrella of advanced biopreservation makes a single governance approach unlikely but coordination essential. Oversight of advanced preservation technologies will require a range of governance frameworks and tools to comprehensively analyze and respond to their cross-sectoral implications. However, advances and challenges in one sector may have implications in others, necessitating coordinated governance approaches.

Scholars of emerging technology have proposed numerous frameworks for analyzing emerging technologies.^40^ This kind of technology anticipation and assessment is prior to and broader than risk assessment in product regulation. Early in technology development, risks may be unclear.^41^ Saner and Marchant note that technology assessment can help build the foundation for later risk specification and regulatory response.^42^ Scholars have noted that responsible innovation utilizes anticipation, reflexivity, inclusion, and responsiveness.^43^ Anticipatory governance uses foresight, engagement, and integration to “act on a variety of inputs to manage emerging knowledge-based technologies while such management is still possible.”^44^ Maynard’s conceptualization of risk innovation “frames risk as a threat to existing or future ‘value’, where value is broadly and multiply defined within personal, societal, and organizational contexts.”^45^ Brass and Sowell have explained that adaptive governance approaches involve iterative processes that respond to emerging risks and problems posed by new technologies.^46^ Tentative governance frameworks similarly recognize the inherent uncertainty of emerging technologies and challenge the benefits of a single, “final” approach to governance.^47^ Mandel has called for “new governance” using “more collaborative, flexible, multi-stakeholder regulatory processes and development” instead of “conventional top-down, ‘command and control’” regulation.^48^ “Midstream modulation” involves internal attempts to analyze and course-correct research and development efforts.^49^

Complex technologies like advanced biopreservation technologies, with a wide range of uses, can mean that “no single entity is capable of fully governing” all of the “multifaceted and rapidly developing fields and the innovative tools and techniques they produce.”^50^ Marchant and Wendell have proposed “Governance Coordination Committees” to integrate and oversee various efforts to regulate broad emerging technologies.^51^ Kuzma and Priest have used “cognate product” and “whole-technology” approaches to analyze the far-reaching implications of nanotechnology.^52^ A cognate-product approach “involves comparing specific products that have already been marketed to similar products of the emerging technology.”^53^ A whole-technology approach “treats the emerging technology as a body of products and methods and relates it to another technological field that has already emerged and penetrated markets.”^54^ Linkov and colleagues have advocated for a “comparative, collaborative, and integrative” risk governance approach for new technologies that combines experimental data and expert insight to shape oversight of complex technologies that pose risks that are difficult to predict.^55^ Trump et al. advocate for a “safety-by-design” approach to governing emerging biotechnology that centers transparency, accountability, participation, integrity, and capacity and applies throughout the technology development lifecycle to prevent unintended consequences of research from reaching across sectors.^56^

The National Academy of Medicine formed the Committee on Emerging Science, Technology, and Innovation (CESTI) to analyze emerging biomedical technologies and “serve as a platform for convening diverse stakeholders… in order to assess governance in health and medicine and drive collective action.”^57^ CESTI developed a “novel governance framework that will enable policymakers to better understand [emerging technologies’] cross-sectoral footprint and anticipate and address the social, legal, ethical, and governance issues they raise.”^58^ The framework connects high-level values such as justice, autonomy, fairness, collective good, and individual good to policy goals and specific policy tools.^59^ In response, Kuzma has argued that current oversight structures lack “spaces for the broader analysis and governance proposed by CESTI,” and has called for the “creation of those spaces.”^60^

In addition to these theoretical frameworks, governance of emerging technologies requires considering both “hard law” and “soft law” options. Hard law “involves standardized governmental rulemaking procedures and outcomes,”^61^ whereas soft law mechanisms are “not directly enforceable” but can “nevertheless create powerful expectations.”^62^ Soft law approaches, which may be initiated by non-governmental entities such as professional societies, may be able to adapt more rapidly than traditional “government-enacted and government enforced regulation,” especially for broad technologies that include a “wide variety of applications, industry sectors, and regulatory authorities.”^63^ Soft law mechanisms are a form of “agile governance” and may enable coordinated international oversight of complex new technologies.^64^

Responsibly developing advanced biopreservation technologies requires developing oversight approaches that address risk and benefit for specific applications, while coordinating governance across applications.

### II. Governance Strategies for Specific Biopreservation Applications

The governance challenge varies by application. Key applications to consider are biopreservation to advance (a) human organ transplantation, (b) tissue banking, (c) development of cell therapies, (d) the study of whole organisms including pathogens, (e) aquaculture, and (f) environmental conservation. The governance challenges analyzed below make clear the need for the kind of consultative, anticipatory, and creative approaches to oversight surveyed above in Part I.

#### A. Organ Transplantation

Advanced biopreservation techniques have the potential to revolutionize the organ transplant system by relieving constraints of time and geography. At present, the maximum storage time for whole organs is measured in hours, and the need for rapid matching means that a “majority of thoracic organs from donors” are never transplanted.^65^ As of January 5, 2024, more than 103,000 people in the US were listed on the organ transplant waitlist and more than 58,000 of those were “active waiting list candidates.”^66^ Enabling storage could save lives.^67^ Advances in perfusion technology are helping to extend the time between organ retrieval and transplant,^68^ but advanced biopreservation could allow for prolonged storage, facilitate better matches, reduce organ nonuse, permit organ quality improvement, and enable donor preconditioning.^69^

The potential for large-scale banking of solid organs raises the question of who would fund and control such banks, and whether they would be operated as a public good by the government or a federal contractor, or instead operated as commercial entities by for-profit companies. These questions are critically relevant as the US organ transplantation system has come under scrutiny based on disparities in organ access and distribution.^70^ Sophisticated organ manipulations will require new supply pathways and more centralized facilities for preservation, storage, and rewarming.^71^ Anticipating and addressing these issues is essential, both in the US and internationally. The rise of large-scale organ banking has potential for great benefit, but risks include commercializing the transplantation system and exacerbating illegal organ trafficking.

In the US, the National Organ Transplant Act (NOTA)^72^ and state statutes following the 2006 Uniform Anatomical Gift Act (UAGA)^73^ prohibit payment for organs, though NOTA does allow for the collection of fees for processing and storage of organs. The resources necessary across the supply and processing pathways that are required for advanced biopreservation and the needed investment by commercial entities may challenge traditional rejection of donor payment and invite commercialization of transplantation.^74^ The rise of biopreservation will call for renewed deliberation on the appropriate role of commercialization in transplantation. It will also call for renewed efforts to combat organ trafficking.^75^

The rise of advanced biopreservation will also pose regulatory challenges. In the US, it remains unclear which regulatory agency should be responsible for ensuring the safety and quality of biopreserved organs. This question is complicated by proposed reforms to organ transplantation at the federal level.

Human organs for transplantation — including vascularized composite allografts (VCAs) like limbs, tracheas, and vascularized limbs — are managed by the Organ Procurement and Transplantation Network (OPTN), under the authority of the Health Resources & Services Administration (HRSA).^76^ For decades, the United Network for Organ Sharing (UNOS) has held the federal contract to operate the OPTN, but the Biden administration has announced plans to “break up the monopoly power” of UNOS.^77^ In March 2023, HRSA announced the OPTN Modernization Initiative to “strengthen accountability, equity, and performance in the organ donation and transplantation FDA may consider regulating biopreserved organs like medical devices, as preserved organs may “resemble analogous devices now subject to regulation.”^83^ The FDA already governs many of the products, devices, and processes involved in organ transplantation.^84^

Within the realm of HCT/Ps, the FDA regulates most intensively tissues that are more than “minimally manipulated.” “[P]reservation for storage and removal from storage” is germane to this determination.^85^ If this processing alters “relevant characteristics” of the tissue, the tissue is no longer considered minimally manipulated and is subject to more stringent oversight.^86^ Organs subjected to advanced preservation techniques including loading of cryoprotective agents (CPAs) and nanomaterials, extended biopreservation, system through a focus on five key areas: technology; data transparency; governance; operations; and quality improvement and innovation.”^78^ The Biden administration has solicited bids to operate the OPTN, “hoping to foster competition in a system that has effectively operated as a monopoly.”^79^ The modernization initiative will “more than double investment in organ procurement and transplantation” and will introduce public-facing “data dashboards” to present “data on organ retrieval, waitlist outcomes, and transplants, and demographic data on organ donation and transplant” for individual transplant centers and organ procurement organizations.^80^

The regulatory landscape is complicated by the question of FDA’s jurisdiction over organs. The FDA’s Center for Biologics Evaluation and Research (CBER) oversees human cells, tissues, and cellular and tissue-based products (HCT/Ps).^81^ However, FDA jurisdiction over HCT/Ps exempts solid organs for human transplant.^82^ The rise of advanced biopreservation will raise the question of whether FDA jurisdiction should be expanded to cover biopreserved organs. Indeed, then laser re-warming followed by offloading of CPAs and nanomaterials may be considered more than minimally manipulated.

Extending the viability of transplantable organs may also allow ex vivo organ modification. This could include genetically modifying organs to improve donor histocompatibility,^87^ boost organ function, or treat genetic disorders. A future that includes genetic modification of human organs and other sophisticated techniques to generate and optimize transplants^88^ would require effective oversight of the organs produced.

Current methods of transplantation are “remarkably safe and effective” and as such there is “little regulatory tolerance for the inherent risks associated with innovation.”^89^ Organs subject to complex biopreservation protocols may undergo changes to physiology and function.^90^ Variations in processing across facilities where organ procurement, manipulation, storage, and transplant take place may introduce further concerns.

Organ biopreservation advances will also necessitate new policies and procedures for organ allocation. Current allocation policies from OPTN and UNOS are molded by limitations of time and geography, among other criteria.^91^ Prolonged organ biopreservation and storage would require reexamination of those allocation rules, as well as new rules to address allocation of biopreserved versus fresh organs. If both are found safe and effective, but one proves more effective (as in the case of IVF with fresh versus frozen embryos), allocation policies will need to consider how to avoid unfairness and inequities in a two-tier system.

#### B. Tissue Banking

Advanced biopreservation may yield benefits by enabling prolonged storage of vascularized tissue, but may exacerbate preexisting issues in tissue banking governance involving quality control and safety. Of the more than three million tissue grafts that are distributed each year, more than two and a half million are transplanted.^92^ Tissue transplants are used for life-saving procedures such as providing skin grafts to patients with severe burn injuries,^93^ as well as for reconstructive surgeries for patients suffering from disease or trauma.^94^

However, monitoring tissue quality for transplant and long-term treatment outcomes has been an issue. Tissue banks must register with the FDA, which has the authority to inspect them.^95^ Few states license and inspect tissue banks.^96^ Tissue processors “are required to report only the most serious adverse events they discover.”^97^ Tracking tissue procurement and transplantation is more difficult than tracking organ transplantation, in part because there is “no central data source” for the volume or frequency of tissue transplants.^98^

Existing US governance also does not require healthcare facilities to track and report on the success or failure of tissue grafts.^99^ FDA regulations require tissue banks to track tissues to a “consignee,” but not to the patient who ultimately receives the tissue.^100^ “Tissue suppliers generally provide information cards for hospitals to complete and return when tissues are implanted” but hospitals do not always cooperate.^101^ Hospitals must have a method in place to track tissue to its final use, but collecting the data is voluntary under FDA regulations and American Association of Tissue Banks (AATB) standards,^102^ and participation varies.^103^

This makes it difficult to track grafts back to a specific donor and to evaluate long-term results. Follow-up data on long-term quality of preserved grafts is critical in determining their effectiveness, but there is no clear mechanism to collect this data. Nor is there an effective mechanism to track contamination. If biopreservation can increase the number of grafts recovered from a donor with a communicable disease, contaminated products may reach more patients. A single donor can provide more than 100 tissue grafts^104^ which may be processed by several tissue banks. Each tissue bank may adopt a unique donor identifier. While the FDA mandates coding to identify tissues, it is “agnostic on what coding system to use” which means that “coding used for tissues is fragmented in the United States.”^105^ Changes may be needed to both FDA requirements and AATB standards to track donor products effectively.

Existing standards, including the FDA tissue bank registration requirements and Good Tissue Practice Requirements as well as the AATB voluntary standards for accreditation, promote safety by imposing requirements on processing.^106^ There is a lack of standardized metrics for assessing the viability and quality of individual tissue grafts. This will make it more difficult to assess the effects of advanced preservation techniques on these grafts. Defining quality in developing such metrics is also crucial. To a tissue bank, a tendon graft may be high quality if it is usable and sterile. However, a recipient may consider a tendon graft to be high quality only if it can perform well for decades. Receipt of FDA approval requires showing that advanced preservation technologies are superior, or at least not inferior to existing tissue transplant methodologies.^107^ Standardized measures of quality are needed.

Advanced biopreservation could be a financial boon for tissue banks, raising questions about the role of informed consent and compensation for donors. At the federal level, tissue banks are regulated by NOTA, which prohibits tissue donors from receiving direct compensation for their tissues but does not bar tissue banks and processors from receiving “reasonable payments” for their services.^108^ A proposed 2007 statute would have required the Secretary of the Department of Health and Human Services (DHHS) to promulgate regulations defining “reasonable payments” for this purpose, but the bill died and the term remains undefined.^109^

Largent notes that “Federal law does not require tissue banks and processors to” operate as nonprofits, and “[t]he U.S. government ‘takes almost no steps’” to prevent tissue processors or banks from earning substantial profits.^110^ Tissues are “routinely commodified” after altruistic donation, and nearly every state treats registration as an organ donor as registration as a tissue donor too.^111^ A single tissue donor can generate up to $220,000 in profit, but their surviving relatives receive none of the financial benefit.^112^ Advanced biopreservation methods could make it easier to store tissues, enabling tissue processors and banks to increase profits. A transition to larger and more profitable tissue banks may raise the question of whether compensating a tissue donor or their family should be allowed.

Advanced biopreservation could also enable creation of large-scale tissue and skin banks for mass trauma events, but questions of who should govern such a resource and under what economic model remain unanswered. Current tissue and skin banks may lack the financial incentive to store large amounts of allograft skin for mass casualty events.^113^ There is informal infrastructure within the AATB Skin Council to coordinate response to a crisis, but the scale of response may be limited.^114^ In the face of a nuclear event in a major US city, for example, current skin banks would be able to provide only about 3% of skin grafts needed.^115^ The Department of Defense (DoD), Defense Advanced Research Projects Agency (DARPA), and US Biomedical Advanced Research Development Authority (BARDA) have recognized this limitation and explored options such as synthetic skin grafts.^116^ Advanced biopreservation techniques could help. However, tissue and skin banks may not be prepared to maintain a massive stockpile, when it is more profitable to respond to ordinary patient demand. Government intervention may be needed to operate this type of bank as a public resource. Such a bank might be incorporated into the existing strategic national stockpile managed by the US interagency Public Health Emergency Medical Countermeasures Enterprise while using the expertise of stakeholders in the AATB.^117^

#### C. Cell Therapies

Applying advanced biopreservation techniques to cell therapies has significant potential to confer clinical and research benefit. For example, preserving islet cells would permit more islet cell transplants, including for patients in remote areas.^118^ Advanced biopreservation may also enable research, as many clinical trials of islet cells have been unable “to culture or store high-quality islets for more than a few days after isolation.”^119^ However, integrating advanced biopreservation in production, storage, and deployment of cell therapies will heighten current regulatory challenges and the need for standards.

Current FDA-approved CAR-T cell therapy products, for example, are primarily created in small batches by manipulating the patient’s own cells to create autografts.^120^ Transitioning to preserved, off-the-shelf allogeneic therapies will require producers to create a new supply chain. The cost and logistics of a cryopreserved cell therapy supply chain are high, which raises questions about accessibility for patients.^121^ Standards and quality measures will be needed across the chain of production, including during biopreservation, to ensure a uniform product. Consultation with stakeholders will be necessary to guide development of products that are affordable and accessible.

Currently, the FDA regulates cell therapies as biologics and requires a lengthy Biological License Application (BLA) process that costs $5–6 million.^122^ When evaluating novel cell therapies, the FDA tends to “err on the side of zero risk.”^123^ The FDA also may require additional testing when adding a cryopreservation or freezing step.^124^ Some researchers have argued that FDA’s vetting process unduly burdens innovation, especially for therapies targeting devastating diseases.^125^ At the same time, the consequences of unsafe cell therapies can be severe and even fatal. In 2021, the FDA halted a clinical trial of allogeneic CAR-T cells after a patient developed chromosomal abnormalities.^126^ Further deliberation may be needed to balance patient protection and life-saving innovation.

The governance challenges extend beyond the United States. While current cell therapy production and use largely occur within a single country,^127^ biopreservation may allow shelf-stable cell therapies to be shipped worldwide. In a 2020 report, the Worldwide Network for Blood and Marrow Transplantation called for “harmonized standards” to create a global regulatory framework.^128^ In 2022, the World Health Organization (WHO) released a report describing a convergent global framework for cell and gene therapy products that would use a “risk-based” approach to evaluation and regulation.^129^ Advanced biopreservation may enable the movement of cell therapies across borders, requiring global standards for production and quality.

#### D. Whole Organisms Including Pathogens

Advanced preservation techniques have the potential to preserve whole organisms ranging in size from single-cell organisms to larger organisms. Enhanced preservation techniques can enable research by improving storage, access to, and quality control of these organisms.

Although the ability to preserve and share model organisms such as zebrafish embryos will be a boon to research, SOPs will be needed. Preservation of aquatic model species may raise reproducibility issues due to a “lack of standardized procedural approaches, lack of standardized terminology, and lack of reporting guidelines.”^130^ Existing repositories of aquatic species, such as the Zebrafish International Resource Center (ZIRC), bank only sperm.^131^ Cryopreserved eggs and embryos will require quality assurance metrics. Third-party organizations such as ZIRC could play a role in establishing these metrics. Journals publishing research on aquatic cryopreservation could require a structured list of information to enhance reproducibility.^132^

Biopreservation can involve inadvertent or deliberate preservation of pathogens. Tiersch and Jenkins note that, “problems posed by cryopreservation basically come from the removal of barriers to travel across distance (frozen samples are more stable and have longer working lifetimes than do fresh samples, and thus can be more widely and easily transferred) and across time (potentially hundreds or thousands of years).”^133^ They address the problem of pathogens (viral, bacterial, fungal, or parasitic) inadvertently preserved in biological specimens. They review state, regional, federal, and international mechanisms to test for pathogens and options to use antibiotics and antifungals prophylactically to treat samples at collection.^134^ However, they recognize that biopreservation will enable biospecimens to be transferred widely, including to those unfamiliar with safety measures, and call for further development of protocols.

Biopreservation can also be used to deliberately preserve pathogens or disease vectors, enabling important research. Biopreservation of the *Cryptosporidium* parasite, for example, is enabling much-needed research that has been hampered by the lack of preservation options and the necessity of maintaining the organism by serial passaging through host animals.^135^ *Cryptosporidium* is estimated to cause thousands of deaths annually plus long-term effects in survivors.^136^ Only one drug is currently approved for treatment and its effectiveness is limited.^137^ Biopreservation of pathogenic organisms can aid creation of standardized reference lines,^138^ promoting access and reproducibility in research.^139^ Cryopreserving pathogens such as *Cryptosporidium* can thus facilitate development of vaccines and treatments.^140^

However, *Cryptosporidium* has been designated by the CDC as a Category B bioterrorism agent.^141^ The parasite is transmissible by waterborne, foodborne, and airborne vectors, causing human and veterinary illness.^142^ Biopreservation, storage, and shipment of the organism will require containment measures and safeguards against accidental or deliberate release. This is especially important in geographical regions where the pathogen is not already endemic. This same concern will arise if the pathogen is released at a future time when resistance has waned or disappeared. Advanced biopreservation with appropriate safeguards and oversight may reduce the likelihood of accidental escape of organisms both in the lab and during transport.^143^ A further protection is that successful rewarming of biopreserved organisms requires sophisticated interventions and expertise.^144^ On the other hand, biopreserved organisms may be more susceptible to neglect as they may require less monitoring and intervention; this could result in forgotten or abandoned organisms, which could fall into the hands of unintended third parties.

Advanced biopreservation could enable access to the pathogen by bad actors interested in attempting bioterrorism.^145^ In response to the terrorist attacks of 9/11, Congress passed The PATRIOT Act of 2001, which prohibits possession of a “biological agent, toxin, or delivery system not reasonably justified by a … research, or other peaceful purpose.”^146^ It imposes a fine, incarceration, or both as penalties for knowing violations.^147^ Additionally, the Bioterrorism Act of 2002 delegated responsibility to DHHS and the Department of Agriculture to establish a list of agents and toxins with “the potential to cause a severe threat to public health and safety” or to animals or plants or their products.^148^ The National Science Advisory Board on Biosecurity (NSABB), has provided guidance on dual-use research of concern (DURC) and the US government has adopted policies and procedures providing heightened review of research protocols raising DURC concerns.^149^

Advanced biopreservation with appropriate safeguards and oversight may actually reduce the likelihood of accidental or deliberate misuse of organisms.^150^ Maintaining the organisms long-term and then successfully rewarming biopreserved organisms so they regain viability requires sophisticated equipment and expertise.^151^ This may limit the potential for accidental or malicious release.

#### E. Aquaculture

Advanced biopreservation has enormous potential to address world-wide shortages in access to aquatic protein sources for adequate diet. Biopreservation could facilitate selective breeding to develop improved lines for aquaculture production systems and could help avoid declines in production by preventing inbreeding depression. However, regulatory approval for aquaculture products created with new technologies (including potentially gene transfer) could be a prolonged process. In the US, the “FDA operates a mandatory safety program for all fish and fishery products.”^152^ In 2015, the FDA approved a genetically engineered salmon for human consumption.^153^ The company had first approached the FDA in the 1990s for approval; officials explained the process was prolonged because this was the “first approval of its kind.”^154^ The FDA’s 2022 guidance for fish products does not address cryopreservation of sperm, eggs, or embryos.^155^ It is unclear at this point if the FDA would regulate aquaculture products created using biopreserved gametes or embryos in the same way it regulates other aquaculture products, or if it would consider these products to be first of their kind and require heightened review. This problem is magnified by the numerous other federal agencies in the US and multiple treaty structures and agreements around the world that address transfer of aquatic organisms as live animals or preserved products.

Public acceptance of biopreserved food is another potential issue. Successful introduction of aquaculture foods that have undergone advanced biopreservation will require learning the lessons of technologies that have prompted public skepticism, such as genetically modified crops. Despite research showing the safety advanced biopreservation within aquaculture will first require development of SOPs for research that not only serve individual laboratories, but that can also be extended directly into application. This would represent a change of focus for traditional research approaches but would offer substantial value through much-increased efficiency. Such efforts would require new approaches and tools to support community-level interaction, first among researchers and then with early adopters and commercial entities. A current lack of standards hinders the translation of biopreservation technologies to small-scale or large-scale commercialization,^163^ and advancements in biopreservation techniques will further complicate this problem. Standardization and harmonization at multiple steps along the technology pipeline will be needed to allow for “commercial-scale application” of biopreservation of genetically modified crops, opposition to GMO foods has been substantial.^156^ On the other hand, use of reproductive technologies in the food chain, including frozen sperm for artificial insemination to produce dairy cows, has been widely accepted. Ascertaining public attitudes can help anticipate concerns (if any) and oversight can aid public confidence. The FDA regulates aquaculture products for quality and safety.^157^ Third-party standards, such as those promulgated by the Aquaculture Stewardship Council, can identify aquaculture producers that use best practices.^158^ Stakeholder outreach that addresses not only facts about the technology, but also cognitive and emotional issues, may avert distrust.^159^

Advanced biopreservation of embryos and gametes for aquaculture could also lead to animal welfare concerns. Large numbers of embryos are needed for these experiments.^160^ Long-term sublethal injuries associated with preservation of gametes and embryos are poorly understood.^161^ Advanced preservation and permeable CPAs may risk epigenetic changes.^162^ More research is needed to fully appreciate the implications of preservation technologies for future generations of aquatic species.

Aquaculture in its current form is a newly developing global enterprise that lags behind the well-established livestock industries in many ways. Applying technologies in aquatic species.^164^

In the short term, technologies for advanced biopreservation can prove expensive, rendering them inaccessible for many users. Commercially available devices for cryopreservation can cost up to $50,000.^165^ An emerging approach is to expand access throughout multiple sectors by developing open hardware devices that can be fabricated, assembled, used, and modified within user communities by sharing digital files over the Internet. Recent advances in 3-dimensional printing, electronics, microprocessors, and software accessibility have brought previously elite capabilities into the consumer market. Powerful technologies are now available as inexpensive equipment (such as 3-D printers costing $250) that only 5–10 years ago were only available to the largest companies and universities. Thus, open-access technologies, including 3D printable cryopreservation hardware, may begin to bridge the access gap.^166^ Such open technology platforms are beginning to be developed to address the “pervasive lack of standardization, affordable hardware, and reproducibility” that limits the scale at which these biopreservation technologies may be applied.^167^ At the root of such development, however, is the need for multidisciplinary and interdisciplinary collaborations. Static departmental organizations may need to be rethought to enable sustained interactions among often siloed groups of engineers, biologists, product designers, software developers, and social scientists to further this movement.

#### F. Environmental Conservation

Advanced biopreservation techniques have the potential to meet urgent conservation needs, but existing environmental laws and regulations may stand in the way. In the US, the federal National Environmental Policy Act (NEPA) mandates environmental impact review for any environmental intervention involving federal land, agencies, or research money.^168^ The federal Endangered Species Act (ESA) prevents “takings” of endangered species, but provides certain exemptions for research use – though getting an exemption can be a long and bureaucratic process.^169^ Globally, the Nagoya Protocol was adopted to promote the “fair and equitable sharing of benefits arising from the utilization of genetic resources.”^170^ The Protocol aims to protect less developed countries from commercial exploitation of resources by foreign commercial interests^171^ and has “reinforced the notion of sovereign rights of nation states over biological resources within their political boundaries.”^172^ Critics of the Protocol, which has been adopted by more than 90 countries, lament the “heavy bureaucratic burden on researchers and their institutions” and worry that host countries may prefer to work with commercial entities for fees rather than share resources with non-commercial researchers.^173^ Conservationists and commercial users alike must “negotiate a prior informed consent agreement and mutually agreed upon terms before” collecting samples from a country that has signed on to the Protocol.^174^ The US is not a signatory, but US researchers must comply with the requirements when collecting samples from countries that have signed on.^175^ A streamlined regulatory framework and process may be needed to enable optimal use of advanced biopreservation in research and conservation.

Advanced biopreservation could be used to build a large-scale biobank as a hedge against ecosystem disaster on earth.^176^ This will require significant funding, oversight, and infrastructure. Advanced biopreservation techniques do not work uniformly across species; identifying species-specific procedures is resource intense.^177^ Once created, maintaining a biobank requires significant monetary investment.^178^ Funding available for non-human biobanks is significantly less than funding for human medical biobanks, and there may be limited support at present to fund biobanks for conservation purposes.^179^

As indicated above, mechanisms for standardization are much needed, including for sharing data and specimens across biobanking.^180^ In the US, there is a substantial effort in agriculture to identify, collect, and catalogue genetic materials from major domesticated livestock breeds.^181^ Without this information, it is difficult to systematically assemble specimens needed for preservation.^182^ The US Department of Agriculture (USDA) National Animal Germplasm Program (NAGP) provides oversight of a massive livestock biobank and has created a comprehensive and publicly accessible database of stored specimens.^183^ The NAGP was funded through the Farm Bill and currently focuses on agricultural species.^184^ Expanding funding and policy could expand the capacity to preserve wild and endangered species.

Advanced biopreservation is a useful tool, but meaningfully protecting biodiversity also requires efforts to preserve animal habitats and ecosystems.^185^ Multiple efforts are under way to use advanced reproductive technologies, cryopreservation, and other technologies to preserve and restore biodiversity.^186^ The effectiveness of artificial reproduction technologies and captive breeding, both of which are facilitated by cryopreservation, has not been established for restoring animals to natural habitats and may have unintended consequences for an ecosystem.^187^ Effective conservation efforts also require engaging with stakeholder beliefs about the significance of animal species. In California, for example, scientists clashed with local Tribes over the acceptability of taking genetic samples from a mountain lion with religious significance.^188^ Conservationists must work with stakeholders to understand the unique needs of the community and ensure that biopreservation is used respectfully.

### III. Building Cross-Cutting Governance for Technology Transition to Advanced Biopreservation

This survey of six major realms in which advanced biopreservation may play a significant role reveals cross-cutting themes. In each domain — spanning human health, the study of whole organisms, food and aquaculture, and environmental conservation — advanced biopreservation techniques have the capacity to do great good. Yet recurrent concerns include the need for oversight in research and deployment; the necessity of standards, metrics, and SOPs to promote predictability, quality, and reproducibility; the requirement for significant investment including in supply chains and facilities; concerns over the source of that investment and control, be it commercial entities, nonprofits, governmental entities, or some combination; and the role of stakeholders and the public.

These cross-cutting themes, despite the disparate domains of potential application for advanced biopreservation technologies, raise the question of whether some kind of cross-domain and cross-sector coordination could help. (See Table 2.) Absent that, each realm in which the technology is applied will face these issues in isolation, raising the possibility of conflicting terminology and standards, inefficient decisional processes that fail to take advantage of analysis and learning in other domains, conflicting policy, and failure to optimize the benefits of biopreservation technology while controlling the risks.

Coordinated governance will first require dialogue across research groups and other stakeholders to build consensus on terminology and standards. Professional associations such as the Society for Cryobiology, or a broad alliance of organizations crossing the anticipated realms in which biopreservation will be applied, could sponsor consensus efforts. Advanced biopreservation is an emerging platform technology with multiple variations and future applications.^189^ As in many areas of innovation, too often research groups operate in isolation and competition. This invites lack of agreement on terminology, unnecessary variation in protocols with resulting barriers to replicability and reproducibility, lost opportunities to build interoperability, and a failure to collaborate to anticipate benefits and harms. Siloed groups also maximize entry barriers to new and less-resourced research groups that would benefit from shared technology. At this upstream point, creating consensus on terminology and measures will support progress by promoting cross-laboratory reproducibility, replicability, and protocols. It will also aid the development of standardized equipment and tools. Indeed, cross-community coordination may invite the development of open-source tools that can be freely accessed.

Second, anticipating the applications of biopreservation discussed above, including both the expected benefits and risks, shows the importance of engaging now with multiple communities. Here again, a cooperating community of researchers and early developers will be better equipped and resourced to undertake public engagement than will multiple research groups operating in isolation. Understanding how different user communities view the possibilities and risks should inform development and application of the technology and will begin to build trust. In organ transplantation, for instance, relevant communities include transplant centers, surgeons, and patients, including those patients who now lack access to transplants and communities historically neglected in engagement efforts. Also important are biobanks and biorepositories, which have long experience in storing and evaluating biospecimens; they may be called upon to play a direct role in the biopreservation pipeline.

Third, the community of early research and developers, with stakeholders from anticipated user and other concerned communities as well as anticipated regulators, should begin and sustain a dialogue on oversight approaches. In some domains of research and application, soft law mechanisms such as consensus guidelines and voluntary agreements may allow development of the technology and understanding of where more regulatory and hard law approaches are needed. Indeed, in the emerging field of nanobiotechnology, Ramachandran and colleagues have argued for “a dynamic oversight approach” that “integrates soft and hard approaches to oversight, moving between these two poles dynamically as data become available and attitudes and analyses evolve,” and “provides for strong coordination among regulatory agencies, stakeholders, and the public.”^190^

Finally, oversight authorities will need to build cross-agency coordination. The need for this is clear in domains such as organ transplantation and environmental conservation — oversight in each of those domains already involves multiple oversight authorities. That crowded landscape invites confusion, conflicting rules, unnecessary overlap, and unwanted regulatory gaps. There is a robust literature on tools for interagency (and even intra-agency) coordination.^191^ Indeed, the Government Accounting Office (GAO) issued a 2023 guide on “Leading Practices.”^192^

## Conclusion

Advanced biopreservation technologies are rapidly emerging. They have the potential to pause biological time by suppressing or temporarily stopping metabolism. By altering time constraints, they can alter geographical constraints as well. These powerful new capabilities offer enormous potential benefits across the six domains we have canvassed: organ transplantation, tissue banking, development of cell therapies, the study of whole organisms including pathogens, aquaculture, and environmental conservation. Yet these new capabilities also pose a wide range of risks, including to health and safety, to environments and ecosystems, and to values such as equity and public accountability.

This article argues that the time to address those risks and benefits is now, when advanced biopreservation technologies are emerging, research is showing success, and the landscape of developers and stakeholders is taking shape. Biopreservation technologies will deeply challenge current governance tools and regulatory agencies in the US The far-reaching potential of these technologies across numerous domains invites confusion, poor decisions on oversight, governance gaps and redundancy, and failure to engage all stakeholders.

We offer four key recommendations to secure a better future. We urge the development of harmonized terminology and standards, sustained engagement across multiple stakeholder communities, dynamic oversight strategically deploying soft law and hard law mechanisms, and deliberate cross-agency coordination. Biopreservation promises to become a keystone technology in advancing human health, feeding the planet’s population, and safeguarding environmental diversity. This advanced technology requires 21st-century governance built on collaboration, consensus, and coordination.

## Figures and Tables

**Table 1:** Major categories of use for advanced biopreservation techniques, and the specific domains of application within each category that are discussed below in Part II. This is not an exhaustive list of all possible uses.

Categories of use	Key domains of application addressed in Part II
Human biomedicine	• Organ transplantation • Tissue banking • Cell therapies
Research on whole organisms	• Model species (e.g., zebrafish) • Pathogens
Food and aquaculture	• Aquatic species
Environmental conservation	• Banking threatened species (e.g., coral) • Translocating organisms for species & ecosystem restoration • Building biorepositories (e.g., a lunar biorepository) and storing genetic resources for research & reproduction

**Table 2:** Four core recommendations for coordinated governance across the expected applications of advanced biopreservation.

Core recommendations for coordinated governance
Dialogue across research groups and stakeholders to build community consensus on terminology and standards.
Early and continuing engagement with expected user groups across anticipated domains of application.
Sustained dialogue across stakeholders on oversight needs and approaches, including the dynamic deployment of “soft law” and “hard law” approaches over time.
Deliberate cross-agency coordination to avoid regulatory confusion, inefficiency, and gaps.
